# A national survey on the implementation of key infection prevention and control structures in German hospitals: results from 736 hospitals conducting the WHO Infection Prevention and Control Assessment Framework (IPCAF)

**DOI:** 10.1186/s13756-019-0532-4

**Published:** 2019-05-08

**Authors:** Seven Johannes Sam Aghdassi, Sonja Hansen, Peter Bischoff, Michael Behnke, Petra Gastmeier

**Affiliations:** 1 Charité – Universitätsmedizin Berlin, corporate member of Freie Universität Berlin, Humboldt-Universität zu Berlin, and Berlin Institute of Health, Institute of Hygiene and Environmental Medicine, Berlin, Germany; 2 National Reference Center for Surveillance of Nosocomial Infections, Berlin, Germany

**Keywords:** Infection prevention, Implementation, Survey, Surveillance, Healthcare-associated infection

## Abstract

**Background:**

Healthcare-associated infections (HAI) pose a burden on healthcare providers worldwide. To prevent HAI and strengthen infection prevention and control (IPC) structures, the WHO has developed a variety of tools and guidelines. Recently, the WHO released the Infection Prevention and Control Assessment Framework (IPCAF), a questionnaire-like tool designed for assessing IPC structures at the facility level. The IPCAF reflects the eight WHO core components of IPC. Data on the implementation of IPC measures in German hospitals are scarce. Therefore, it was our objective to utilize the IPCAF in order to gather information on the current state of IPC implementation in German hospitals, as well as to promote the IPCAF to a broad audience.

**Methods:**

The National Reference Center for Surveillance of Nosocomial Infections (NRZ) sent a translated version of the IPCAF to 1472 acute care hospitals in Germany. Data entry and transfer to the NRZ was done electronically between October and December 2018. The IPCAF was conceived in a way that depending on the selected answers a score was calculated, with 0 being the lowest possible and 800 the highest possible score. Depending on the overall score, the IPCAF allocated hospitals to four different “IPC levels”: inadequate, basic, intermediate, and advanced.

**Results:**

A total of 736 hospitals provided a complete dataset and were included in the data analysis. The overall median score of all hospitals was 690, which corresponded to an advanced level of IPC. Only three hospitals (0.4%) fell into the category “basic”, with 111 hospitals (15.1%) being “intermediate” and 622 hospitals (84.5%) being “advanced”. In no case was the category “inadequate” allocated. More profound differences were found between the respective core components. Components on multimodal strategies and workload, staffing, ward design and bed occupancy revealed the lowest scores.

**Conclusions:**

IPC key aspects in general are well established in Germany. Potentials for improvement were identified particularly with regard to workload and staffing. Insufficient implementation of multimodal strategies was found to be another relevant deficit. Our survey represents a successful attempt at promoting the IPCAF and encouraging hospitals to utilize WHO tools for self-assessment.

**Electronic supplementary material:**

The online version of this article (10.1186/s13756-019-0532-4) contains supplementary material, which is available to authorized users.

## Background

Healthcare-associated infections (HAI) pose one of the most severe threats to the health of patients and remain a challenge for healthcare providers worldwide [1]. A recent point prevalence survey conducted in 28 EU-countries and Serbia revealed an estimated prevalence of patients with HAI in acute care hospitals of 6.5% [2]. When extrapolating prevalence data to estimate the burden of HAI on the healthcare system, it is estimated that over 2.6 million HAI occur annually in the EU. Further extrapolations suggest that these HAI account for a total of 501 disability-adjusted life years (DALYs) per 100,000 general population and an attributable number of over 90,000 deaths per year [3]. Although these figures solely apply to the European context, various studies have illustrated that HAI are also a problem in healthcare settings outside the EU, particularly in low- and middle-income countries [4]. Accordingly, institutions such as the World Health Organization (WHO) and others have in the past placed a high emphasis on developing and promoting strategies to prevent HAI [5–7].

As one of their key documents to strengthen infection prevention and control (IPC) aspects, the WHO has released the “Guidelines on Core Components of Infection Prevention and Control Programmes” offering countries as well as individual healthcare facilities an orientation on how to establish and strengthen IPC activities [8]. At the facility level, the WHO distinguishes between eight core components (CC), which address different aspects of IPC. These are:

- IPC program (CC1)
- IPC guidelines (CC2)
- IPC education (CC3)
- HAI surveillance (CC4)
- Multimodal strategies (CC5)
- Monitoring/audit of IPC practices and feedback (CC6)
- Workload, staffing and bed occupancy (CC7)
- Environments, materials and equipment for IPC (CC8)

To facilitate the establishment of IPC structures, the WHO has released manuals giving advice on how to implement the WHO Guidelines on Core Components of Infection Prevention and Control Programmes at a national and at a facility level [9]. Especially at the facility level, implementation of IPC key aspects differs widely, not only between countries of different income levels, but also within countries themselves [10–13]. Therefore, to provide healthcare facilities with an additional tool to assess, analyze and improve IPC activities at their facilities, the WHO has recently released the Infection Prevention and Control Assessment Framework (IPCAF) [14]. In form of a questionnaire, facilities can answer questions relating to IPC with the objective to determine strengths and weaknesses.

Previous studies have investigated the applicability and feasibility of other WHO tools, such as the WHO Hand Hygiene Self-Assessment Framework and the multimodal approach at hand hygiene in general [15–18], as well as the Water and sanitation for health facility improvement tool (WASH FIT) [19]. Studies have repeatedly demonstrated the feasibility and reliability of these tools and approaches. Due to the recent release of the IPCAF, the tool has not yet been applied on an equally broad range as the tools mentioned above.

In 2011, the German Protection against Infection Act (“*Infektionsschutzgesetz*”) was revised, augmenting the importance of IPC in hospitals. However, data on the implementation of IPC measures and structures in German hospitals are scarce. Thus, it was our objective to describe the current state of implementation of key IPC aspects, as defined by the WHO core components, in German hospitals using the IPCAF. Additionally, we wanted to promote the IPCAF to motivate hospitals in Germany to perform an IPC self-assessment.

## Methods

In Germany, HAI surveillance is well established. Over 2000 hospitals have participated (i.e. at least temporarily provided data) in the German nosocomial infection surveillance system “KISS” (*Krankenhaus-Infektions-Surveillance-System*) since its establishment in the 1990s. Annually, surveys are sent to the participating hospitals to address topics of current interest, which are relevant within the context of surveillance and HAI prevention in Germany.

The National Reference Centre for Surveillance of Nosocomial Infections (NRZ) in Germany translated the IPCAF into German. On the first of October 2018 the translated version was sent to 1472 acute care hospitals in Germany as the annual KISS-survey in the form of a link to a survey webpage, into which all data were entered. Data entry was possible until the end of December 2018. Table e1 to be found in the Additional file 1 illustrates structural characteristics of these 1472 hospitals. Participation was on a voluntary basis. After completing the survey, the results were automatically transferred to the NRZ. The received data were not linked to surveillance data or other data such as alcoholic hand rub consumption of the participating hospitals due to privacy and data protection regulations. The translated version of the IPCAF can be found in the Additional file 2 of this article.

As mentioned above, the IPCAF was conceived in a way that individual questions on IPC aspects had to be answered. Every possible answer of a question was allocated a score. Following the concept of the eight core components of IPC, the IPCAF was divided into eight sections. For every core component the scores of the individual questions were aggregated. A maximum score per core component of 100 was possible. The final IPCAF score was calculated by adding the scores of all eight core components (i.e. maximum total score possible was 800). Depending on the final score, the hospitals were grouped into four different IPC categories:

- 0–200 points: inadequate
- 201–400 points: basic
- 401–600 points: intermediate
- 601–800 points: advanced

After reception of the data, the NRZ conducted a descriptive analysis for the total IPCAF score, as well as for the scores of the respective core components and for some selected individual questions of particular interest.

All data were anonymized and collected in accordance with paragraph 23 of the German federal law, German Protection against Infection Act (“*Infektionsschutzgesetz*”), which regulates the prevention and control of infectious diseases in humans. Therefore, ethical approval and informed consent were not required.

## Results

**Fig. 1:**
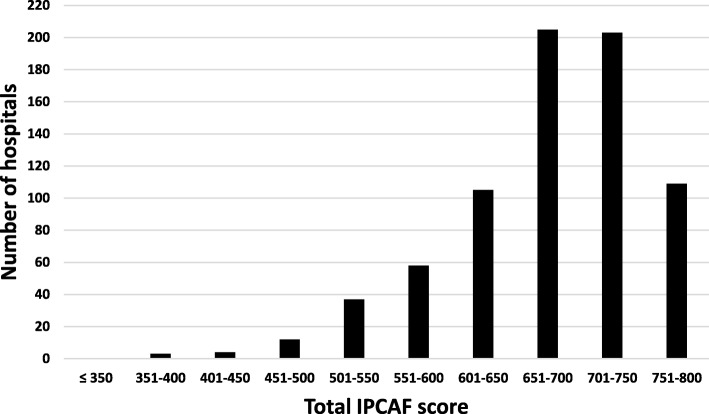
Distribution of the total IPCAF score among participating hospitals. Legend: *IPCAF* Infection Prevention and Control Assessment Framework

Altogether, 739 hospitals (response rate of 50.2%) conducted the IPCAF and transferred data to the NRZ. Of these datasets, three were incomplete and therefore excluded from all analyses. As a result, data from a total of 736 hospitals were included and further analyzed. The overall median score, which was attributed to the participating hospitals, was 690, with an interquartile range between 640 and 730. When grouped by score into the above-mentioned IPC categories, only three hospitals (0.4%) fell into the category “basic”, with 111 hospitals (15.1%) being “intermediate” and 622 hospitals (84.5%) being “advanced”. In no case was the category “inadequate” (less than 201 points) allocated. Figure 1 illustrates the distribution of scores among participating hospitals.

Differences were found with regard to the score of the individual components. Table 1 illustrates the mean, median, first and third quartile, as well as the tenth and 90th percentile for the overall IPCAF score and for the scores of each individual CC. CC7 with its focus on workload, staffing, ward design and bed occupancy had the lowest median score (75), while CC5 (multimodal strategies) had the lowest mean score (71). Guidelines (CC2) and environment/infrastructure (CC8) were the two components with the highest scores. The median score of CC2 was 100 and the mean score of CC8 was 96. The range of scores per component, defined as the range between the tenth and the 90th percentile was broadest for the component on multimodal strategies (CC5) and narrowest for environment/infrastructure (CC8).

**Table 1:** Distribution of results of the total IPCAF score and scores per core component

Component	Score					
	Q10	Q25	Q50	Q75	Q90	Mean
CC1	67.5	80	88.8	95	100	85.7
CC2	87.5	94.4	100	100	100	95.7
CC3	65	75	85	90	100	82.7
CC4	72.5	85	92.5	97.5	100	88.9
CC5	40	60	80	90	95	71.3
CC6	62.5	75	85	92.5	97.5	82.7
CC7	45	60	75	90	95	74.1
CC8	90	95	97.5	100	100	96.1
Total	575	640	690	730	762.5	677.3

*IPCAF* Infection Prevention and Control Assessment Framework, *CC* core component, *Q10* tenth percentile, *Q25* first quartile, *Q50* median, *Q75* third quartile, *Q90* 90th percentile

A deeper examination of the IPCAF dataset into the level of individual questions and answering patterns yielded diverse results. Due to the complexity of the IPCAF, we cannot illustrate all of these results in this article, but will instead focus on selected topics of particular interest and with a relatively large variability between hospitals. For the purpose of conciseness, we will primarily look at the components with the lowest scores, which were multimodal strategies (CC5) and workload/staffing (CC7). The majority of hospitals (645; 88%) reported that multimodal strategies were utilized to implement IPC interventions. However, only 541 hospitals (74%) stated that an interdisciplinary team was involved in implementing these interventions and 545 hospitals (74%) reported involving staff designated for quality and patient-safety improvement in order to conceive and promote multimodal strategies. Bundles and checklists were found to be rather prevalent, with 625 hospitals (85%) using them as an integral part of their multimodal strategies. Further results of CC5 per multimodal strategy are shown in Table 2.

**Table 2:** Results per multimodal strategy from IPCAF core component 5

Element	Answer	Number (%)
System change	Element not included in multimodal strategies	111 (15.1)
	Interventions to ensure the necessary infrastructure and continuous availability of supplies are in place	218 (29.6)
	Interventions to ensure the necessary infrastructure and continuous availability of supplies are in place and addressing ergonomics and accessibility, such as the best placement of central venous catheter set and tray	407 (55.3)
Education and training	Element not included in multimodal strategies	32 (4.3)
	Written information and/or oral instruction and/or e-learning only	439 (59.6)
	Additional interactive training sessions (includes simulation and/or bedside training)	265 (36.0)
Monitoring and feedback	Element not included in multimodal strategies	83 (11.3)
	Monitoring compliance with process or outcome indicators (for example, audits of hand hygiene or catheter practices)	183 (24.9)
	Monitoring compliance and providing timely feedback of monitoring results to health care workers and key players	470 (63.9)
Communications and reminders	Element not included in multimodal strategies	124 (16.8)
	Reminders, posters, or other advocacy/awareness-raising tools to promote the intervention	385 (52.3)
	Additional methods/initiatives to improve team communication across units and disciplines (for example, by establishing regular case conferences and feedback rounds)	227 (30.8)
Safety climate and culture change	Element not included in multimodal strategies	257 (34.9)
	Managers/leaders show visible support and act as champions and role models, promoting an adaptive approach and strengthening a culture that supports IPC, patient safety and quality	321 (43.6)
	Additionally, teams and individuals are empowered so that they perceive ownership of the intervention (for example, by participatory feedback rounds)	158 (21.5)

*IPCAF* Infection Prevention and Control Assessment Framework

In CC7, staffing, ward design and bed occupancy aspects were addressed. Two thirds of the participating hospitals (490) reported conducting an assessment of staffing needs at the facility using national or international standards. Correspondingly, 255 hospitals (35%) stated that they did not have a system in place to react to results of a staffing needs assessment, and only 234 hospitals (32%) stated that they maintained a defined healthcare worker to patient ratio at all times in the entire facility. Additional results of CC7 on ward design and bed occupancy are illustrated in Table 3, along with other questions of particular relevance and interest from other core components.

**Table 3:** Selected results of the IPCAF from various core components

Topic	Answer	Number (%)
Existence of IPC program (CC1)	Not existent	32 (4.3)
	Existent but no clearly defined objectives	262 (35.6)
	Existent with clearly defined objectives and annual activity plan	442 (60.1)
Defined IPC objectives in critical areas (CC1)	No IPC objectives	43 (5.8)
	IPC objectives only	137 (18.6)
	IPC objectives and measurable outcome indicators	285 (38.7)
	IPC objectives and measurable outcome indicators and future targets	271 (36.8)
Senior facility leadership (CC1)	Does not provide specific allocated budget	230 (31.3)
	Provides specific allocated budget	506 (68.8)
Senior facility leadership (CC1)	Does not show demonstrable support	213 (28.9)
	Shows demonstrable support	523 (71.1)
IPC training of healthcare-workers (CC3)	Not existent	1 (0.1)
	Only in written and/or oral and/or online form	437 (59.4)
	Interactive training (e.g. bedside teaching)	298 (40.5)
IPC training and training of other specialties (CC3)	IPC aspects not integrated into training of other specialties	146 (19.8)
	IPC aspects integrated into training of some other specialties	302 (41.0)
	IPC aspects integrated into training of all other specialties	288 (39.1)
Feedback of surveillance data (CC4)	No annual feedback	8 (1.1)
	Annual feedback in written and/or oral form only	237 (32.2)
	Annual feedback via presentation and interactive problem-solution finding	491 (66.7)
Ward design (CC7)	Not in accordance with international standards	90 (12.2)
	Certain departments in accordance with international standards	199 (27.0)
	All departments in accordance with international standards	447 (60.7)
Patient placement in corridor beds outside the room (CC7)	More frequently than twice a week	61 (8.3)
	Less frequently than twice a week	162 (22.0)
	Never	513 (69.7)

*IPCAF* Infection Prevention and Control Assessment Framework, *IPC* infection prevention and control, *CC* core component

A full description of all questions and the answers we received from the participating hospitals can be found in the Additional file 3 of this article.

## Discussion

To our best knowledge, our survey represents one of the first applications of the IPCAF on a broad scale. By making use of the existing surveillance structures in Germany, we were able to distribute a translated version of the IPCAF to a large number of recipients and thereby generate a great amount of data. The primary conclusion we can draw from the data received, is that in general IPC structures and activities are well established in Germany. Collectively, the participating hospitals reached a median score of 690, which by the definitions applied in the IPCAF, translated to an advanced IPC level. However, with some hospitals falling into the categories basic or intermediate, our survey demonstrated a certain degree of heterogeneity and a potential for improvement. With regard to the individual components of the IPCAF, we found substantial differences between the respective scores. The first core component focuses on the existence and characteristics of an IPC program. The median score of 89 revealed that IPC programs are generally widely established in Germany. This could be interpreted as a result of the reform of the German Protection against Infection Act, which increased the awareness of IPC aspects in the country. Nevertheless, when focusing on specific questions of CC1, more diverse results were seen. For instance, around 40% of hospitals stated that their IPC program lacked clearly defined objectives and less than 40% of hospitals reported having defined future targets for their IPC program. Along with the non-optimal results concerning IPC staffing and the lack of support from the senior facility leadership in a substantial proportion of hospitals (Table 3), these results illustrate a remarkable potential for improvement.

Scores for IPC guidelines (CC2) and IPC training and education (CC3) were generally high with few exceptions. Potential for improvement, however, can be found with reference to the methods applied to perform IPC training. Only around 40% of hospitals reported utilizing interactive teaching methods (e.g. bedside training), which have been proven in the past to be an effective form of education [20, 21]. However, this finding corresponds with data from other fields of medicine, which see a decline in the application of this didactic method [22, 23]. Moreover, IPC education could also be improved by implementing IPC aspects into the training of other specialties of medicine, which currently less than 40% of hospitals seem to undertake systematically (Table 3).

Multimodal strategies, which are the main topic of the fifth core component, are a relatively new concept in the practice of infection control [15, 24, 25]. We saw a mean score of only 71 in this component, which illustrated a clear deficit. CC5 yielded the most diverse results of all components, indicating that this rather novel approach at IPC is already implemented in a considerable number of German hospitals, and yet, still represents a relevant potential for improvement. This was especially true for questions on the individual elements of multimodal strategies, such as system change, education and training, etc. (Table 2).

HAI surveillance (CC4), as well as monitoring and auditing of IPC processes (CC6), were revealed to be well established in German hospitals. As demonstrated in many publications, Germany has a well-functioning surveillance network with a long history [26]. Timely and appropriate feedback of surveillance data is one of the key aspects of conducting successful surveillance [27]. Our survey, however, suggested a deficit concerning the way that surveillance data is fed back in many German hospitals. A third of participating hospitals reported giving no feedback at all or in written/oral form only, not embracing a more interactive approach (Table 3).

With the eighth core component investigating structures such as water and electricity supply, it is obvious that this component is more geared towards low- or middle-income settings, thus not being fully applicable to the German context. Unsurprisingly, scores for this component were generally very high.

Understaffing has previously been demonstrated to be a risk for the occurrence of HAI [28, 29]. Therefore, the deficits found to exist with regard to CC7, which focuses, among other aspects, on the healthcare worker to patient ratio as a key aspect of IPC, gain relevance. Less than a third of all hospitals recorded maintaining a defined healthcare worker to patient ratio in the entire facility at all times. Remarkably, around a third of all hospitals, reportedly, were not conducting a staffing needs assessment using national or international standards and had no system in place to react to a change in the demand for staff. These findings confirm the previously described shortage of qualified staff for patient care in German hospitals [30] and represents one of the most relevant findings of our survey. Along with the deficits for ward design and bed occupancy, illustrated in Table 3, we can conclude that improvements in the field of workload and staffing are of urgent need in German hospitals.

When interpreting the data generated through this survey several limitations have to be recognized. Among the most relevant were:

- In spite of numerous footnotes and explanations provided, the IPCAF required a profound understanding of the WHO terminology and underlying concepts. Relatively new concepts, such as multimodal strategies, were not understood by every respondent, leaving room for misinterpretation and false reporting.
- The IPCAF collected information, which, although handled discreetly by the NRZ, may have been perceived as potentially compromising by some hospitals. Therefore, in some cases questions could have been answered wrongly purposefully to achieve a higher score.
- Facilities with a high interest in aspects of IPC may have had a greater interest in completing the survey (overall response rate of 50.2%) and may therefore be overrepresented.
- Due to the electronic form of data entry and anonymization of data, participants were not able to retroactively correct data entry mistakes once the survey was completed and data sent to the NRZ. This may explain some unexpected individual results.
- The IPCAF did not collect information such as hospital size, type or ownership, which would be helpful to better interpret some of the data generated.

Besides these limitations, our survey had numerous strengths. The most relevant were:

- A high number of hospitals participated, which allowed for careful extrapolations to the national level.
- Germany has a long history of surveillance, which increased the understanding of many concepts addressed by the IPCAF and the readiness of hospitals to participate in the survey.
- The translation of the survey into German allowed participants to answer the IPCAF in their native language, thereby reducing the language barrier.
- The NRZ provided help and advice for hospitals that had difficulties interpreting certain questions.

## Conclusion

IPC structures and processes are in general well established in Germany. In particular, this can be concluded for IPC guidelines and HAI surveillance. Conversely, a potential for improvement was discovered especially with regard to the implementation of multimodal strategies and for aspects of workload and appropriate healthcare worker staffing. To our best knowledge, our survey represents the first broad application of the IPCAF, and will serve as a useful orientation for future applications within Germany and in other countries. Developments and trends may become apparent through repeated application of the IPCAF. The primary purpose of the IPCAF was to enable healthcare facilities to perform an IPC self-assessment. Lessons learned from the IPCAF by the participating hospitals (e.g. through identifying deficits) may reveal themselves in years to come in form of activities to strengthen IPC structures.

## Additional files

Additional file 1: Structural characteristics of 1472 German acute care hospitals invited to participate in the WHO Infection Prevention and control assessment framework (IPCAF). (DOCX 12 kb)

Additional file 2: Infection Prevention and control assessment framework. (IPCAF) (German translation). (DOCX 82 kb)

Additional file 3: Results of the Infection Prevention and Control Assessment Framework (IPCAF) in 736 German hospitals. (DOCX 45 kb)

## Abbreviations

CC: Core component
DALYs: Disability-adjusted life years
HAI: Healthcare-associated infection(s)
IPC: Infection prevention and control
IPCAF: Infection Prevention and Control Assessment Framework
KISS: Krankenhaus-Infektions-Surveillance-System
NRZ: National Reference Center for Surveillance of Nosocomial Infections
WASH FIT: Water and sanitation for health facility improvement tool
WHO: World Health Organization
