# Supplementary material for: A national survey on the implementation of key infection prevention and control structures in German hospitals: results from 736 hospitals conducting the WHO Infection Prevention and Control Assessment Framework (IPCAF)
Source: Antimicrob Resist Infect Control. 2019 May 8;8:73. doi: 10.1186/s13756-019-0532-4 (PMC6505265; doi:10.1186/s13756-019-0532-4)
Supplement: Supplementary file 1 — Structural characteristics of 1472 German acute care hospitals invited to participate in the WHO Infection Prevention and control assessment framework (IPCAF). (DOCX 12 kb) [file 13756_2019_532_MOESM1_ESM.docx]

| **Parameter** | **Group** | **Value** or  **Number (percentage)** |
| --- | --- | --- |
| Hospital size (i.e. number of hospital beds) | First quartile | 136 |
|  | Median | 240 |
|  | Third quartile | 420 |
| Hospital type | Primary care | 295 (20) |
|  | Secondary care | 508 (35) |
|  | Tertiary care | 180 (12) |
|  | Maximum care (incl. university hospitals) | 154 (10) |
|  | Specialized hospital | 222 (15) |
|  | Other/Unknown | 113 (8) |
| Hospital ownership | Public (not further specified) | 469 (32) |
|  | Private (not further specified) | 334 (23) |
|  | Not for profit (public and private) | 168 (11) |
|  | Ecclesiastical | 367 (25) |
|  | Other/Unknown | 134 (9) |

**Table e1** Structural characteristics of 1,472 German acute care hospitals invited to participate in the WHO Infection Prevention and Control Assessment Framework (IPCAF)
